# Foundations under pressure: Qualitative interviews on the impact of prolonged pandemic and public health measures on Ontario youth and young adults

**DOI:** 10.1371/journal.pone.0307423

**Published:** 2025-10-06

**Authors:** Laurel C. Austin, Makayla Nunes Gomes, Sebastian Chavez, Celina Degano

**Affiliations:** 1 Ivey Business School, Western University, London, Ontario, Canada; 2 Health Sciences, Western University, London, Ontario, Canada; 3 Faculty of Information and Media Studies, Western University, London, Ontario, Canada; 4 Public Health Ontario, Toronto, Ontario, Canada; Touro University California College of Pharmacy, UNITED STATES OF AMERICA

## Abstract

**Introduction:**

Foundations of youth and young adults’ (YYAs) lives were shaken by the prolonged COVID-19 pandemic. This study explores how over a year of pandemic, with several periods of stay-at-home orders, impacted Ontario YYAs and important foundations that influence their development and future wellbeing.

**Methods:**

Semi-structured interviews were conducted April – June 2021 during stay-at-home orders. Nineteen Ontario YYAs, age 16–21, were asked how relationships, education, work, and important events had been impacted by the pandemic and public health responses, and how they had coped. Reflexive thematic analysis was used to iteratively engage with data to distill themes. Respondents included: eight high school students, ten post-secondary students, one full time employed.

**Results:**

Three main themes were identified. First, disruptions to individual agency and life course (theme), included (subthemes): drastic shift to increased time and isolation at home; feeling responsible for others’ health and safety; learning and professional development suffered; precarious employment; and lost beginnings and endings to important transitions. Second, relationships and connectedness changed (theme), included (subthemes): Friendships were altered; family dynamics changed; and international students faced unique challenges. Third, wellbeing and coping (theme), included (sub-themes): stress was high; coping methods were diverse; and connecting digitally was suboptimal. Some friendships were lost; some grew closer. Those in their last year of high school/university in spring 2020 or 2021 lost important transitional endings (graduation, etc.). Those starting post-secondary in fall 2020 also lost transitional beginnings (e.g., leaving home, on-campus experience, meeting new friends).

**Conclusions:**

There is need for further research to assess long-term impacts, especially among YYAs who had family members at severe risk and those who graduated high school during the pandemic. Public health practitioners should work with high schools and post-secondary schools to develop approaches to limit the severity of altered schooling and important transitions in future upheavals.

**“** *It feels like we’re in an alternate dimension and the world is so chaotic…*

*how is this not a movie*?” (Respondent in study)

## Introduction

After SARS-CoV-2 (aka COVID-19) was declared a global pandemic by World Health Organization (WHO) on March 11, 2020, governments world-wide implemented public health measures to limit viral spread including social distancing, school closures, travel restrictions, “lockdowns” and more [1]. These brought drastic changes to foundations that shape youth and young adults’ (YYAs’) lives and futures. Friendships, social interactions, and growing independence influence personal identity [2–4]. Friends and parents help young people in decision making [5,6] and friends ease transitions to new phases of life [5]. Important life events, e.g., graduation and first year of post-secondary school, mark transitions to adulthood [2,4]. Quality of education impacts future education, employment, and wellbeing [7,8]. Early work experiences offer skill development and networks for future employment [9–11].

COVID-19 was first global pandemic since the Spanish Flu (started in 1918). Qualitative research allows rich exploration of new phenomenon, but became less feasible during COVID-19 due to need to modify research methods with new ethics considerations [12,13]. This is evidenced in a review of 168 studies from around the world on YYA pandemic school experiences [14] – only 12% employed qualitative methods, including those using one/few open-ended survey questions.

A handful qualitative studies early in the pandemic reported on how the pandemic impacted YYAs broadly [15–19]. But the impacts of a prolonged pandemic might well differ from those early in pandemic, during initial lockdowns. Yet, we’ve identified only one qualitative study, from Norway, on how long-lasting regulations of the *prolonged* pandemic broadly impacted YYAs and their quality of life. Using focus groups a year into the pandemic, they found that quality of life had steeply declined, largely due to impacts on schooling, socializing, and missed life events [20]. Other qualitative studies late in the pandemic, also small in number, employed specific theoretical lenses, often mental health [21,22]; examined impacts on specific sub-populations, (e.g., homeless, disabled, Indigenous) [22–25]; or focused on specific topics, e.g., changes in schooling [26,27] or impacts of self-isolating [28].

While it is important to look deeply into specific impacts of prolonged pandemic, as most studies late in pandemic did, it is also important to understand broadly, the multi-faceted impacts individuals experienced. This cannot be deduced from impersonal surveys that examine statistical relationships, but by talking to YYAs about their lives. The purpose of this study was to explore, using individual interviews, how the prolonged pandemic that had lasted over a year with several periods under stay-at-home orders impacted Ontario YYAs’ lives and the important foundations they build upon, including relationships with family and friends, education, employment, and important events.

## Context

Ontario had three periods with province-wide stay-at-home orders between March 2020 and June 2021, with the most densely populated areas under such orders for up to eight months in total [29]. It was Canada’s province with the most weeks of in-person school closures during COVID-19 [30]. Throughout the 2020–2021 school year high schools varied learning approaches (virtual, in-person, hybrid), and schedules, extracurriculars, etc. as local case counts changed [31]. Post-secondary schools were online March 2020 to at least August 2021 [29].

## Methods

### Study design

A qualitative descriptive approach was used, appropriate when the objective is to provide a summary of participants’ beliefs and experiences in their own words when time and resources are limited [32]. Semi-structured interviews were used. This method has advantages over surveys, which do not allow probing to better understand context or perceptions, and over focus groups that risk having one or few participants sway discussion and thus data.

Respondents were asked how the pandemic that had started over a year earlier had impacted their life, followed with prompts as needed regarding: impacts on school, employment, relationships; missed important events, and how they had coped. These were initial questions in interviews that went on examine YYA vaping decisions as part of a broader research program. As a result, our sample can be considered a convenience sample. To aid the reader in assessing rigor we followed the consolidated criteria for reporting qualitative studies (COREQ) [33]. Research ethics approval was granted by Western University Research Ethics Board (REB). Interviews were conducted virtually by two researchers (MG, SC) over Zoom (n = 18) or phone (n = 1). With consent, interviews were recorded and verbatim transcripts produced and verified (n = 18) or detailed notes taken (n = 1). Participants received a $30 gift card.

### Participants and recruiting

We planned to interview 20 YYAs, greater than the 12–15 needed to elicit the most common beliefs in a broader population [34]. Advertisements on Facebook/Instagram recruited participants during March – May 2021, stating the research was “to understand how the pandemic has affected youth and young adults’ lives and activities”. Those who clicked on the ad were directed to a pre-screening survey. The pre-screen was completed by 235 YYAs, each entered into a draw for one of four $40 gift cards; 85 (36%) met the inclusion criteria of age 16–21, in Ontario, and having ever vaped (per broader study). As such, our sample over-represents YYAs who ever vaped. Interviews were conducted April to June 2021. Purposeful sampling maximized diversity in the sample.

### Data analysis

Reflexive thematic analysis [35–38] was used to analyze transcripts, acknowledging that themes are not objective findings, but derived from researchers’ iterative engagement with, and subjective interpretation of verbal data. We iteratively engaged with the data, searching for patterns and shared meaning among responses, developing themes that convey what we took away given intense interaction with data. Through initial reading by SC, MNG, and LCA we identified a preliminary set of concepts and themes and corresponding codes. Three authors (MNG, LCA, CD) read the transcripts repeatedly to consider individual context and further develop themes and codes. MNG and LCA then coded transcripts in detail using MaxQDA software to code utterances. The authors met several times to suggest, discuss, and refine themes. Participants did not provide feedback on findings.

### Research team reflexivity

Our team included an undergraduate student (SC) and master’s student (MNG) experiencing the pandemic in ways similar to respondents, thus well-suited to build rapport with them. They conducted/transcribed interviews and were involved in analysis and manuscript preparation. CD brings experience and research expertise as a public health professional with an MPH and experience in youth development and engagement. LCA is a Behavioral Decision Theorist (PhD) with experience in mixed methods adolescent and adult decision-making research.

## Results

Interviews averaged 32 minutes. Twelve respondents self-identified as female, seven male (none selected ‘other’ or ‘do not wish to disclose’). Eight were in high school, ten post-secondary school, one full-time employed. Nine self-identified as White, four as East, South or Southeast Asian, one as Latin American, one as Middle Eastern, four as ‘mixed-race’; fourteen lived in urban/city areas, four in suburbs, one rural. One respondent (age 20) made some nonsensical statements, then expressed concern about being overheard. That interview was ended early and excluded. We have organized findings into three main themes: disruptions to individual agency and life course; relationships and connectedness; wellbeing and coping. Among these we’ve identified eleven sub-themes shown in Table 1.

**Table 1:** Identified main and subthemes.

Main Themes	Subthemes
T.1 Disruptions to individual agency and life course	T1.1 Drastic shift to increased time and isolation at home T1.2. Feeling responsible for others’ health and safety T1.3 Learning and professional development suffered T1.4 Precarious employment T1.5 Lost beginnings and endings to important transitions
T.2 Relationships and connectedness changed	T2.1 Friendships were altered T2.2 Family dynamics changed T2.3 International students faced unique challenges
T.3 Wellbeing and coping	T3.1 Stress was high T3.2 Coping methods were diverse T3.3 Connecting digitally was suboptimal

### Theme 1: Disruptions to individual agency and life events

Disruptions to individual agency and life events reflects significant impacts on routines, responsibility, control over own life, and important transitions to a next phase in life. This theme includes five subthemes: (T1.1) Drastic shift to increased time and isolation at home; (T1.2) Feeling responsible for others’ health and safety; (T1.3) Learning and professional development suffered; (T1.4) Precarious employment, and (T1.5) Lost beginnings and endings to important transitions.

#### T1.1 Drastic shift to increased time and isolation at home.

Prior to the pandemic respondents were seldom home, instead at school, work, in extra-curriculars, and out with friends. This all changed: respondents described having been, “*strictly at home*”, “*in my room all day*”, and “*stuck in the basement*”. Many post-secondary students had unexpectedly moved back to parents’ homes early in the pandemic; one respondent lamented having to *“learn how to be a child again”(P03, 21, 2*^*nd*^ *Year Post-Secondary)*. Only one, in a rural area, and who did not socialize a lot pre-pandemic shared *“[not being] all that restricted*” (*P10, 20, Employed*).

Few questioned necessity of restrictions. Several expressed confusion and anger towards peers who broke rules and partied, often commenting about social media posts. Some celebrated news the current public health restriction would end soon: *“I’m like, ‘Oh, we might not get to just be at home anymore, I might have to actually go to work’. So I have moments where I’m excited” (P13, 19, 1*^*st*^ *Year Post-Secondary).*

#### T1.2 Feeling responsible for others’ health and safety.

Respondents were not concerned about contracting COVID-19 themselves, saying that they were young, healthy, would only have flu-like symptoms, etc. Many did, however, discuss fears of passing COVID-19 to others, not wanting related *‘blame,’ ‘burden,’ ‘embarrassment,’* or *‘having it on my conscious.’* Most reported worry about higher-risk loved ones getting COVID-19, describing these as a senior, grandparent, pregnant, with an auto-immune or chronic condition, currently smokes cigarettes. They were “*worried*”, “*stressed*”, “*terrified*” that more vulnerable individuals would contract COVID-19, possibly from them. A few believed COVID-19 would be fatal for parents and described protective actions taken: “*As soon as I was able to get the vaccine, I did” (P03, 21, 2*^*nd*^ *Year Post-Secondary)*, and: *“If [my mom] gets it, she’s not gonna make it… I’ve been avoiding six feet pretty much at all costs” (P09, 16, High School).*

#### T1.3 Learning and professional development suffered.

While one suggested her school had highly involved parents and was functioning well, most respondents discussed negative impacts to education and learning. Communication with teachers/professors, guidance counselors, staff, and other students was less possible, disjointed, and unsatisfying: “*I have to email the teachers and hope that they reply within a couple of days….I feel I learned better being able to… sit there and interact with students” (P11, 19, 1*^*st*^ *Year Post-Secondary)*. Participants also expressed difficulties with loss of educational accommodations, e.g., rather than having a quiet exam space provided at school, online exams were in the midst of home distractions.

While a couple expressed satisfaction with virtual learning, the general consensus was that it negatively impacted learning. A high school student attributed lack of motivation to the monotony of online education: “*As soon as we would get hit with the high case numbers and we’d go to all online…it’d be the same thing, every day: just waking up, going to my computer, going back to bed. It’s monotonous.”(P07, 17, High School).* Even some with online learning pre-pandemic and who expressed preferring online learning were frustrated with delivery during the pandemic, although one reported liking the move to virtual *“because I like self-paced learning” (P17, 17, High School).*

Educational concerns extended to changes in hands-on professional training. A nursing student shared, *“[M]y evaluations are supposed to be in-person, clinical…it was a virtual clinical experience, every Friday, 8-12 hours listening to an instructor,”* and worried about impacts on skills *(P20, 21, 3*^*rd*^ *Year Post-Secondary)*. Another shared, *“I wasn’t able to go on that [Army Reserve] course, which has held back my training by about a year.” (P07, 17, High School).* Suspended co-ops/internships meant lost opportunities to build skills and networks, causing concern about future opportunities among those affected. Co-ops that took place were virtual and offered limited opportunity for hands-on work, interpersonal interaction, and mentorship.

#### T1.4 Precarious employment.

Only two respondents had never been employed; one was seeking employment, the other wanted to work to pay for further education but lived with immune-compromised grandparents and so could not. Among those currently working, most had had periods of reduced hours or unemployment during the past year. Many had lost or quit jobs due to pandemic and had found or were seeking new opportunities. A couple mentioned feeling the need to earn their own income: “*I haven’t gotten a lot of responses, unfortunately, but I haven’t applied to a lot of places… both my parents lost their jobs, so I felt, like, in an awkward position to,... have to deal with money” (P04, 17, High School).* Some valued interaction with others at work: *“It’s like, ‘Oh yes, I get to work this week, I get to get out of the house finally’. And then it’s like, ‘Oh no you don’t.’ But I will say, when I do get go and work, talk to my mentor, it’s a really good opportunity” (P09, 16, High School).*

#### T1.5 Lost beginnings and endings to important transitions.

Delayed or missed endings associated with transitions into young adulthood were painful losses for those completing high school (n = 5) or post-secondary (n = 1) during the pandemic. When discussing missed events, one said, *“I’m missing out on graduation. I’m going to go pick up my diploma with my two friends, which sucks. …” (P14, 21, 4*^*th*^ *Year Post-secondary)*. A high school student said, *“[I missed] my entire high school. I’m not getting a graduation ceremony… all the major events in my high school career haven’t happened: dances, fundraisers, events, assemblies, none of that” (P16, 17, High School).*

Three who had missed high school endings in spring 2020 also missed transitional beginnings and important events during post-secondary school that fall, including living on-campus, freshman orientation, university classrooms, meeting new friends. One said, “*I’ve missed graduation, prom, everything. Being able to go into first year classes*” *(P11, 19, 1st Year Post-Secondary).* Another discussed how everything had changed, yet did not seem to: *“I went from Zoom High School to Zoom University, which to me seems the same, but…it was much heavier, the educational aspect.* M*entall*y *it was overwhelming” (P13, 19, 1*^*st*^ *Year Post-Secondary).*

### Theme 2: Relationships and connectedness changed

This theme reflects that respondents reported a variety of ways important relationships and respondent connectedness with others were impacted, composed of three sub-themes: (T2.1) Friendships were altered; (T2.2) Family dynamics changed; (T2.3) International students faced unique challenges.

#### T2.1. Friendships were altered.

All respondents discussed friendship impacts. Pain from separation was palpable, especially among high school respondents: *“I’ve definitely seen people outside of my family significantly less, like pretty much not at all” (P17, 17, High School*); “*Some of my friends I haven’t seen in over a year” (P09, 16, High School).* Some reported having lost friends: *“My closest friends, I see less, but still somewhat consistently. It’s more acquaintances I’ve fallen out of touch with … I mean the circle has to be kept small”* (P06, 17, High School). Some discussed becoming closer to some friends, *“A lot of my friends, I lost them, but I got really close with two or three people” (P09, 16, High School).* Many discussed how time together had changed, meeting in smaller groups, walking or riding bikes outdoors.

Respondents highlighted similar challenges maintaining relationships with significant others. A few mentioned that their boy/girlfriend was the only, or one of the few people they had spent time with. We heard of delayed first romance because being together in-person would not be possible, that moving back to their parents’ home interrupted dating relationships, and that trying to maintain relationships via video calls or only outdoors was difficult.

First-year post-secondary respondents were disappointed from the unrealized opportunity to make new friends. “*My first year I didn’t really get to meet anybody. It’s kind of just made everything kind of lonely” (P11, 19, 1*^*st*^ *Year Post-Secondary).* Those most likely to report seeing friends as they had pre-pandemic were post-secondary students living independently, usually with roommates. One international student reported moving away from roommates who didn’t follow pandemic guidelines.

#### T2.2. Family dynamics changed.

Respondents’ families and relationships were impacted in many ways. Stress arose from being housebound together, inability to travel to see local grandparents and distant relatives, parent(s)’ job loss, deaths, worsened pre-pandemic stressful situations, and parental divorce. One attributed “*family conflict*” to being “*cooped up*” and parents having no time alone. Another reported that stress caused by a parent’s pre-pandemic health concerns was worsened, to the point, *“I was afraid that it was going to dissolve our family.”* This respondent went on to say, *“I lost one of my grandparents because of COVID-19. So you know, this disease has been very difficult on me, my family” (P03, 21, 2*^*nd*^ *Year Post-Secondary).* One whose family was concerned COVID-19 would be fatal for their mother described how this led to separation of immediate family: *“…my dad said, [given concerns about mother] ‘even though the law says we can go back and forth [between divorced parents’ homes] - let’s just stop’… So I wasn’t seeing see my dad, wasn’t seeing my sister…”(P09, 16, High School).*

Some participants reported improved family relationships. One said,*“[my sister and I] got closer because we connected more” (P20, 21, 3*^*rd*^ *Year Post-Secondary)*. Another said, *“At the beginning [my mother and I] fought a lot, because we weren’t used to being home all the time… now I would say we’re closer and get along well” (P01, 17, High School).*

#### T2.3 International students faced unique challenges.

Respondents included two post-secondary international students. One shared challenges of family separation for an extended time. *“I’ve not really been able to see my family, since the beginning of the pandemic because of travel restrictions and that’s been really taking a huge toll on my mental health…..Last Christmas, I completely shut off from my parents. I was like, ‘I do not want to talk to you guys, this is awful, talking to you is only going to make me sad’….But I would describe it as healthy now because I feel like I get ample space, but I can also Facetime them [anytime]” (P12, 21, 3*^*rd*^ *Year Post-Secondary).*

The other discussed the solitude of quarantine: “*… [the university said], ‘leave [residences] after one week,’ so it was very hectic … I went home [to Asia]…when I came back it got really bad because over here…there was the stay-at-home order. It was pretty boring, and by law, I had to quarantine for two weeks”* (*P19, 20, 2*^*nd*^ *Year Post-Secondary)*.

### T3. Wellbeing and coping

This theme relates to how respondents were impacted by all of the changes and the approaches they used to cope. This theme is composed of three sub-themes: (T3.1) Stress levels were high, (T3.2) Coping strategies were diverse, and (T3.3) Connecting digitally was suboptimal*.*

#### T3.1. Stress was high.

Although we did not ask about mental health, most respondents talked about “*stress”,* and several mentioned *“anxiety*” or impacts on “*mental health*”. Stress and boredom resulted from “*monotonous,*” “*repetitive,*” days that “*melted together*”. Pre-existing mental health conditions were said to have worsened, and new diagnoses received. Respondents were “*lonely”* and missed friends. They were “*overwhelmed*” and had “*lost motivation”* to learn, socialize, or leave the house: “*My mental health has definitely taken a big hit because I’ve gotten so used to just not doing anything. Even the smallest bit of interaction is really draining now” (P17, 17, High School).* Another reflected “*[I]t’s taken such a bad mental toll on me that I, it almost felt like I was in purgatory at that time” (P12, 21, 3*^*rd*^ *Year Post-Secondary).* Another expressed, “*After a certain point, you just have to start laughing at it, you know, because if you don’t laugh at it, you’ll cry about it. So which one do you want to do” (P15, 20, 3*^*rd*^ *Year Post-Secondary)*.

#### T3.2 Coping strategies were diverse.

When discussing how they coped, some talked about going for walks with friends, discovering new activities/hobbies, or exercising more: *“I picked up a lot more hobbies…. I’ve been reading and writing like crazy” (P16, 17, High School).* One had a very structured approach*: “I use an Excel [worksheet] and make a schedule…I’ve done too much work, I’m going to go for a bike ride. Setting myself goals. I’m trying to hit 10K in 30 minutes, which I hit the other day, and it was the first time I felt euphoric in a long time” (P09, 16, High School).* Another discussed that their physical education class and teacher had helped them cope with pandemic stress.

Some mentioned substance use for coping. *“Just smoke some weed, every night, just to have the time pass by” (P15, 20, 3*^*rd*^ *Year Post-Secondary).* Another said, *“…. I started smoking [cannabis] a lot more often….I’m basically stagnant at home, so why not go and smoke…. A positive one for me is I also got a lot more exercise” (P20, 21, 3*^*rd*^ *Year Post-Secondary).*

#### T3.3 Connecting digitally was suboptimal.

Responses revealed many uses of digital technology to communicate, socialize, and collaborate during the pandemic. We heard of socializing via Netflix and Discord parties, online gaming, and virtually consuming substances with friends. Extra-curricular activities such as band and dance competitions, formal events like graduation ceremonies, and post-secondary orientation became virtual. Respondents reported that “*maintaining friends*” online was not especially fulfilling: “*Yeah, phone calls and Facetime, like that’s all good, but like at a certain point it almost feels like a physiological need to have that in-person interaction*”*(P15, 20, 3*^*rd*^ *Year University).*

This challenging time provided students the opportunity to be creative and seek out innovative ways to connect. One first year post-secondary respondent developed a Discord server to support connecting, socializing and studying with peers. They were really proud of this work and felt that it was helpful to many, reporting over 500 users. Still, respondents expressed that virtual togetherness is sub-optimal: *“I don’t think any of my friends participated in frosh online, because it was like there’s no point….it’s not the same as physical orientation where you’re like, ‘Hey I wanna do this, do you want to be friend?” (P13, 19, 1*^*st*^ *Year Post-Secondary).*

## Discussion

In summarizing what we heard, the word “loss” is a good descriptor. A wide body of literature shows that people feel losses more than equivalent gains and that we are deeply “loss averse” [39]. While early pandemic studies reported YYAs missed time with friends [15,19] and feared losing touch with friends [19], many in our study reported they *had* *lost* friends during the pandemic. If there was a silver lining, it’s that several discussed growing closer to some friends. We heard of other losses: time with friends, freedom, in-person learning, jobs, professional development, roommates, activities, grandparents, holidays with family, and more.

Like previous studies [15,19,27] this study revealed the pain of missing high school graduation. Our work suggests that missing university graduation was equally difficult. Unlike those other studies, this study was able to explore the impacts of the pandemic on the transition from high school to post-secondary school. Three of our respondents lost a traditional high school graduation in spring 2020 and began post-secondary school that fall, sharing they had also “lost” their first year of university. Research shows that how an event is remembered is determined by how a person felt during the best or worst time during that event or based on how it ended [40,41]. This may suggest the pandemic especially tainted memory of high school and post-secondary education for these YYAs. Further research is needed to study the long-term impacts of these losses, especially among this already vulnerable group of youth transiting into young adulthood. This research can inform decision-making and policies to better support youth and young adult health, wellbeing, and education in future pandemics or other major upheavals. 

A second descriptor emerging from the interviews is “protector.” A study early in pandemic found that a small percentage of YYAs mentioned “constant worry” about family members getting COVID-19 when asked about challenges they faced [19]. We found on-going, daily concern for loved ones perceived as high-risk to be prevalent. We cannot generalize from our small sample, but a reasonable portion of our respondents believed COVID-19 would be very serious, even fatal, for someone they lived with. None questioned the need for protective actions they took, but the responsibility weighed heavily. Further research is needed to understand what additional supports this cohort of YYAs may need in the present and in the future to best support them in response to this unique and challenging experience.

Lacking relevant expertise, we did not set out to study mental health. Yet, unprompted, several of our respondents explicitly discussed “mental health” or “anxiety” impacts, and many more discussed stress. There was extensive study of YAA mental health during the pandemic, most of it conducted in early months, most using quantitative methods to assess correlated factors [42]. Our descriptive qualitative research adds rich insight into the complexity of factors Ontario YYAs experienced during the prolonged pandemic, how impacts accumulated and compounded over a year, and confirms that YYAs themselves perceived their mental health to be impacted. The interviews provided insight into how this population was impacted, how they felt, and how they coped with this unique and challenging time in history.

## Conclusions and limitations

There was only a short window in 2020−2021 to do research during the height of the global COVID-19 pandemic. This work adds to a very small body of qualitative research that captured, in YYAs’ own words how they and several important foundations in their lives had been affected by the prolonged pandemic. It is an unusual study in that we conducted individual interviews that allowed probing for beliefs, experiences, and reflections in ways surveys, even those asking open-ended questions, or focus groups could not. Our study is also unique in that our sample included YYAs who were transitioning from adolescence into adulthood, a period characterized by a range of cognitive, emotional, social and neurological changes. This period involves key life events, such as high school graduation, leaving home, start of post-secondary education or a career, all of which had been disrupted for this YYA cohort during the COVID-19 pandemic.

Our study is limited by use of a small convenience sample recruited via social media, and over-representing YYAs who had ever-vaped. Either of these could introduce unknown bias into our study. However, we have no reason to expect that YYAs who had ever-vaped, including those who currently vaped, differed systematically from other YYAs in how they experienced the pandemic given the high prevalence of having ever-vaped in Canada – 29% of age 15–19 and 48% of 20–24 in 2021 [43]. Similar arguments, we believe, are true regarding YYAs who did or did not use social media. As with other small qualitative studies, our results cannot be generalized to the broad population of high school and post-secondary students in spring 2021. This does not diminish that discussions were rich and illuminating, sometimes evoked strong emotions, and shed light on individual contexts that shaped experiences and informed beliefs, adding depth and nuance to current understanding of the complex ways that YYAs were affected by the COVID-19 pandemic.

While some of our respondents really struggled, others were able to cope and used the opportunity to explore new hobbies, get more physically active and saw improvements in some close relationships. This study provides some insights into YYA experiences, feelings, behaviour and adapted coping strategies employed during a prolonged pandemic period of high uncertainty and frequent change.
